# Neuroprotective Effects of TRPM7 Deletion in Parvalbumin GABAergic vs. Glutamatergic Neurons following Ischemia

**DOI:** 10.3390/cells11071178

**Published:** 2022-03-31

**Authors:** Pengju Zhang, Wei Li, Yaan Liu, Yanqin Gao, Nashat Abumaria

**Affiliations:** State Key Laboratory of Medical Neurobiology and MOE Frontiers Center for Brain Science, Institutes of Brain Science, Fudan University, Shanghai 200032, China; 16111520034@fudan.edu.cn (P.Z.); leewei@fudan.edu.cn (W.L.); 16111010003@fudan.edu.cn (Y.L.)

**Keywords:** TRPM7, brain ischemia, parvalbumin neurons, oxidative stress, Akt

## Abstract

Oxidative stress induced by brain ischemia upregulates transient receptor potential melastatin-like-7 (TRPM7) expression and currents, which could contribute to neurotoxicity and cell death. Accordingly, suppression of TRPM7 reduces neuronal death, tissue damage and motor deficits. However, the neuroprotective effects of TRPM7 suppression in different cell types have not been investigated. Here, we found that induction of ischemia resulted in loss of parvalbumin (PV) gamma-aminobutyric acid (GABAergic) neurons more than Ca^2+^/calmodulin-kinase II (CaMKII) glutamatergic neurons in the mouse cortex. Furthermore, brain ischemia increased TRPM7 expression in PV neurons more than that in CaMKII neurons. We generated two lines of conditional knockout mice of TRPM7 in GABAergic PV neurons (PV-TRPM7^−/−^) and in glutamatergic neurons (CaMKII-TRPM7^−/−^). Following exposure to brain ischemia, we found that deleting TRPM7 reduced the infarct volume in both lines of transgenic mice. However, the volume in PV-TRPM7^−/−^ mice was more significantly lower than that in the control group. Neuronal survival of both GABAergic and glutamatergic neurons was increased in PV-TRPM7^−/−^ mice; meanwhile, only glutamatergic neurons were protected in CaMKII-TRPM7^−/−^. At the behavioral level, only PV-TRPM7^−/−^ mice exhibited significant reductions in neurological and motor deficits. Inflammatory mediators such as GFAP, Iba1 and TNF-α were suppressed in PV-TRPM7^−/−^ more than in CaMKII-TRPM7^−/−^. Mechanistically, p53 and cleaved caspase-3 were reduced in both groups, but the reduction in PV-TRPM7^−/−^ mice was more than that in CaMKII-TRPM7^−/−^ following ischemia. Upstream from these signaling molecules, the Akt anti-oxidative stress signaling was activated only in PV-TRPM7^−/−^ mice. Therefore, deleting TRPM7 in GABAergic PV neurons might have stronger neuroprotective effects against ischemia pathologies than doing so in glutamatergic neurons.

## 1. Introduction

Transient receptor potential melastatin-like-7 (TRPM7) is an ion channel protein with a serine-threonine kinase domain in its C-terminal, which is involved in various physiological and pathological processes [1,2]. In neural systems, TRPM7 ion channel and/or its kinase domain have been shown to regulate synaptic density, plasticity and memory [3] as well as synaptic terminal function and neurotransmitter release [4,5]. Earlier investigations revealed that oxygen–glucose deprivation increases TRPM7 conductance in cortical neurons and that blocking or suppressing TRPM7 reduces cortical neuronal death [6]. TRPM7 expression is increased following focal cerebral ischemia in rats [7]. Suppression of TRPM7 expression (by shRNA) in the rat hippocampus reduces neuronal death, cognitive impairment and motor deficits [8]. Treatment with carvacrol, an inhibitor of the TRPM7 ion channel, decreases neuronal degeneration, microglial activation and oxidative stress damage following global cerebral ischemia in rats [9]. Therefore, global inhibition or suppression of TRPM7 in neural systems exerts neuroprotective effects against ischemia. However, the neuroprotective effects of TRPM7 suppression in different cell types have not been investigated.

Brain ischemia in rats increases glutamate and reduces GABA release [10]. Meanwhile, thrombolytic treatment is believed to promote recovery by increasing GABA and reducing glutamate release during acute brain ischemic injury [11]. Studies show that reduction in GABAergic synaptic transmission contributes to the upregulation in overall excitatory activity in the ischemic brain, which results in neuronal death [12]. Parvalbumin (PV) neurons are the most abundant GABAergic neurons in the cortex [13,14]. Studies show that GABAergic PV neurons are more vulnerable to brain ischemia damage than glutamatergic neurons [15] (but also see [16]). Therefore, glutamatergic and GABAergic neurons have different contributions to/sensitivity from ischemia pathologies. 

Despite the clear neuroprotective effects of TRPM7 suppression, its protective role in GABAergic vs. glutamatergic neurons has not been addressed. In the current study, we compared the neuroprotective effects of TRPM7 deletion in GABAergic PV neurons with that in glutamatergic neurons. To achieve our aim, we generated two lines of conditional knockout mice, with deletion of TRPM7 in PV neurons and in CaMKII neurons and subjected them to brain ischemia followed by behavioral, histological, cellular and biochemical analyses.

## 2. Materials and Methods

### 2.1. Experimental Animals

All the experiments involving animals were conducted in accordance with the guidelines of the Fudan University Committee on Animal Care and Use (license number: SYXK-2020-0032). We induced conditional TRPM7 knockout in mice as we described before [3]. Briefly, homozygous TRPM7-floxed mice (TRPM7^flox/flox^) (2–5 months of age) were crossed with CaMKII-Cre mice (2–5 months of age) or PV-Cre mice (2–5 months of age) to generate mice with brain-specific complete deletion of TRPM7, namely, in the CaMKII-positive glutamatergic neurons (CaMKII-TRPM7^−/−^) or PV-positive GABAergic neurons (PV-TRPM7^−/−^). All experimental mice were group-housed (3–5 per cage) with ad libitum access to food and water under controlled conditions: room temperature (23 ± 2 °C), humidity (55 ± 5%), and 12:12 h reversed light–dark cycle (light onset at 20:00). Behavioral experiments were performed during the dark phase. All TRPM7^flox/flox^ mice were used for control mice. Data were collected from 2- to 3-month-old male mice.

### 2.2. Middle Cerebral Artery Occlusion (MCAO) Models

For the development of MCAO models, all experimental mice (body weight of 23–28 g) were randomly assigned to a sham or brain ischemia group. Briefly, mice were anesthetized with 1.5% isoflurane in a 30% O_2_/70% N_2_ mixture under a spontaneous breathing state. The left carotid arteries were exposed and isolated from the branches, and the left external carotid artery (ECA) was ligated and cut approximately 1.5 mm from the bifurcation. A nylon monofilament coated with a silicone tip was then inserted into the left ECA and advanced along the left internal carotid artery (ICA) to occlude the middle cerebral artery. Sixty minutes later, the monofilament was withdrawn to produce reperfusion. The rectal temperature of mice was controlled at 37 °C ± 0.5 °C using a temperature-regulated heating pad during surgery. Regional cerebral blood flow (CBF) was detected in all MCAO mice with Laser Doppler Flowmetry (PF5000, PERIMED, Järfälla, Sweden) to confirm the induction of ischemia. During MCAO, mice that did not reach a CBF reduction of 75% of the pre-ischemia baseline levels were excluded from the experiments. The sham group mice underwent the same anesthesia and surgical procedures except for the middle cerebral artery occlusion.

### 2.3. Immunofluorescence Imaging and Quantitative Analysis

Seven days postoperatively, the experimental mice were sacrificed under anesthesia with 2% isoflurane and perfused with 4% paraformaldehyde. After gradient dehydration with 10%, 20%, and 30% sucrose, brain tissues were embedded in an optimum cutting temperature compound (OCT) tissue-freezing medium. Then, 25 μm frozen sections were prepared. All sections were blocked with blocking solution (5% goat serum, Solarbio, Beijing, China and 0.3% Triton X-100, Sangon, Shanghai, China) for 90 min at room temperature. Next, the sections were incubated overnight at 4 °C with the following antibodies: primary anti-PV (Mouse, 1:200, Merck, Darmstadt, Germany), anti-CaMKIIα (Rabbit, 1:200, Novus, Centennial, CO, USA), anti-CaMKIIα (Mouse, 1:100, Cell Signaling Technology, Danvers, MA, USA), anti-TRPM7 (Rabbit, 1:100, Alomone, Israel), anti-neuronal nuclei (NeuN) (Rabbit, 1:500, Abcam, Cambridge, UK), anti-glial fibrillary acidic protein (GFAP) (Mouse, 1:500, Merck), anti-ionized calcium-binding adaptor molecule 1 (Iba1) (Rabbit, 1:1000, Wako, Richmond, VA, USA), anti-tumor necrosis factor alpha (TNF-α) (Rabbit, 1:1000, Invitrogen, Waltham, MA, USA), anti-phospho-Akt (Ser473) (Rabbit, 1:100, Cell Signaling), anti-p53 (Mouse, 1:200, Cell Signaling), and anti-cleaved caspase-3 (Rabbit, 1:400, Cell Signaling), respectively. After washing with 0.01 M PBS, sections were incubated with CF-488 (1:500, Biotium, Fremont, CA, USA), −555 (1:500, Biotium), or −647 (1:500, Biotium) conjugated secondary antibody for 2 h at room temperature. Further incubation with DAPI (1:1000, Merck) was performed at room temperature for 10 min, and the stained sections were photographed using FV1000 or VS120 microscopy systems (Olympus, Shinjuku, Japan). For full description of microscopic, optical and imaging conditions, see Table 1.

Quantification of infarct size was conducted by using NeuN immunostaining as described before [17]. Briefly, we prepared ten sections with 300 μm intervals for each mouse. Infarct areas were estimated using an indirect method to avoid tissue swelling or shrinkage using the following equation: contralateral hemisphere area–undamaged ipsilateral hemisphere area. For all other immunostaining quantitative analyses, three sections were prepared for each protein from each mouse of the six mice included in each of the groups. Two to three fields (Table 1) were selected in the ischemia penumbra of the cortex for each section. For cleaved caspase-3 immunostaining quantitative analysis, three fields were selected in the ischemia core of the cortex for each section because there were no obvious cleaved caspase-3 positive signals in the penumbra of the cortex. However, due to loss of brain tissue in the ischemia core of the cortex, unequal numbers of cleaved caspase-3 immunostaining images were obtained in the three MCAO groups.

To quantify the number of cells in the optical field, Image J software (v1.53, NIH, Bethesda, MD, USA) was used. The cell density was then calculated as number of cells/mm^2^. To quantify the florescent signal of TRPM7 immunostaining, Image-Pro Plus (v6.0.0.260, Media Cybernetics, Rockville, MD, USA) was used. All brain sections were stained, imaged and analyzed together under same conditions. Individual cell bodies were outlined, special filters were added to calibrate the background to the same level in all images and then the average of optical density of TRPM7 fluorescent signal within the cell body was calculated. For TNF-α quantitative analysis we applied a similar procedure to that used for TRPM7 analysis, but the quantification of the fluorescent signal was conducted in the entire optical field.

### 2.4. Determining the Core, Penumbra and Imaging Areas

Except for the infarct areas (Bregma 1 to −1.72 mm), all our quantitative analyses were done on brain sections taken from Bregma 0.5 to 1.1 mm. All images were taken within primary somatosensory cortex or primary motor cortex. We used the core/penumbra boundary as our reference for taking the images as follows: most of the data were obtained from images taken 0–800 μm from the boundary. P53 images were right across the boundary line. Caspase data were obtained from images taken 0–800 μm from the boundary, but within the core. The cortical core/penumbra boundary was determined by the obvious disruption in structure and morphology of the targeted cortical area as observed under the microscope. For capsase-3 and p53 quantitative analysis, we combined these experiments with NeuN staining to help determining the boundary and core area precisely.

### 2.5. Neurological Deficit Score

The neurological deficit score was evaluated at 2, 4, and 6 days post-MCAO, as described before [18,19]. Briefly, the score was defined as follows: 0 point, no observable deficit; 1 point, failure to extend fully the right forepaw; 2 points, the grip strength of the right forepaw is weakened; 3 points, circling to the contralateral side if the tail was hold, but moved to any direction if the tail was not hold; 4 points, circling to the contralateral side regardless of whether the tail was hold or not; 5 points, moved only after stimulation; 6 points, no response to stimulation and low level of consciousness; 7 points, death.

### 2.6. Foot Fault Test

The foot fault test was commenced at 2, 4, and 6 days post-MCAO. Foot fault errors were scored and calculated as described before [17,20]. Briefly, mice were placed on an elevated (by 30 cm) grid surface (L 40 × W 20 cm). Area of each of the grid openings was 4 cm^2^. During the movement on the grid, the number of the foot faults made by the right forelimb and the steps of right forelimb were counted. Each test consisted of three trials, 1 min each, with an interval of 1 min between trials. The foot faults were expressed as a percentage of the number of errors made by the right forelimb out of the right forelimb total steps.

### 2.7. Western Blot

Seven days after brain ischemia, cortical tissues from the penumbra were harvested and homogenized in RIPA buffer (Beyotime, Nantong, China) containing protease and phosphatase inhibitors (Roche, Penzberg, Germany). The homogenized tissue was centrifuged at 12,000 rpm for 15 min. The supernatants were collected and stored at −80 °C until use.

Equal amounts of proteins were resolved by 10–15% SDS-PAGE gel and transferred to PVDF membrane. After blocking with 5% skim milk, the membranes were incubated overnight at 4 °C with one of the following primary antibodies: anti-GFAP (Mouse, 1:1000, Merck), anti-Iba1 (Rabbit, 1:1000, Wako), anti-TNF-α (Rabbit, 1:1000, Invitrogen), anti-p-Akt (Rabbit, 1:1000, Cell Signaling), anti-Akt (Mouse, 1:500, Merck), anti-p53 (Mouse, 1:1000, Cell Signaling), anti-cleaved Caspase-3 (Rabbit, 1:1000, Cell Signaling) and anti-β-actin (Mouse, 1:10,000, Cell Signaling), respectively. After washing with PBS, membranes were incubated with anti-rabbit (1:5000, Biotium) or anti-mouse (1:5000, Biotium) HRP-conjugated secondary antibodies for 2 h at room temperature. Then, the membranes were incubated with enhanced chemiluminescence solution (Tanon, Shanghai, China). The images were captured by the Tanon 5200 multi-imaging system and quantified with Gel Pro Analyzer software (v4.0.00.001, Media Cybernetics).

### 2.8. Enzyme Linked Immunosorbent Assay (ELISA)

Cortical tissue from the penumbra from the same animals used for Western blot experiments were harvested and homogenized in cold PBS containing protease and phosphatase inhibitors. Then, the homogenized tissue was centrifuged at 12,000 rpm for 15 min. The supernatants were collected and stored at −80 °C until use. TNF-α concentration was detected by using the Mouse TNF-α ELISA Kit (Biolegend, San Diego, CA, USA) according to the manufacturer’s instructions.

### 2.9. Statistical Analysis

Minimum sample size was determined by power calculation using GPower software (V3.1.9.4, Franz Faul, Kiel, Germany). All other statistical analyses were performed by using Prism (GraphPad v5.01, San Diego, CA, USA). The Shapiro–Wilk normality test was used to check if the data were normally distributed. For two-group experiments, we assessed variance homogeneity by using the F test. For multiple-group experiments, the Brown–Forsythe test was used for variance homogeneity. Detailed statistical results of normality and variance tests for all datasets are summarized in the Supplementary Materials (Supplementary Table S1). For comparison between two groups, the two-tailed unpaired *t*-test (normally distributed data), unpaired *t*-test with Welch’s correction (variance not homogenous) or Mann–Whitney test (not normally distributed) was used depending on the normality and variance homogeneity of the data. For comparison among multi groups: one- or two-way ANOVA followed by Bonferroni’s post-hoc test (normally distributed) or one-way ANOVA Kruskal–Wallis test followed by Benjamini post-hoc (not normally distributed) test was used. All data are presented as mean ± SEM. A *p*-value < 0.05 was considered statistically significant.

## 3. Results

### 3.1. Brain Ischemia Induces a Greater Loss of PV Neurons than of CaMKII Neurons

**Figure 1:**
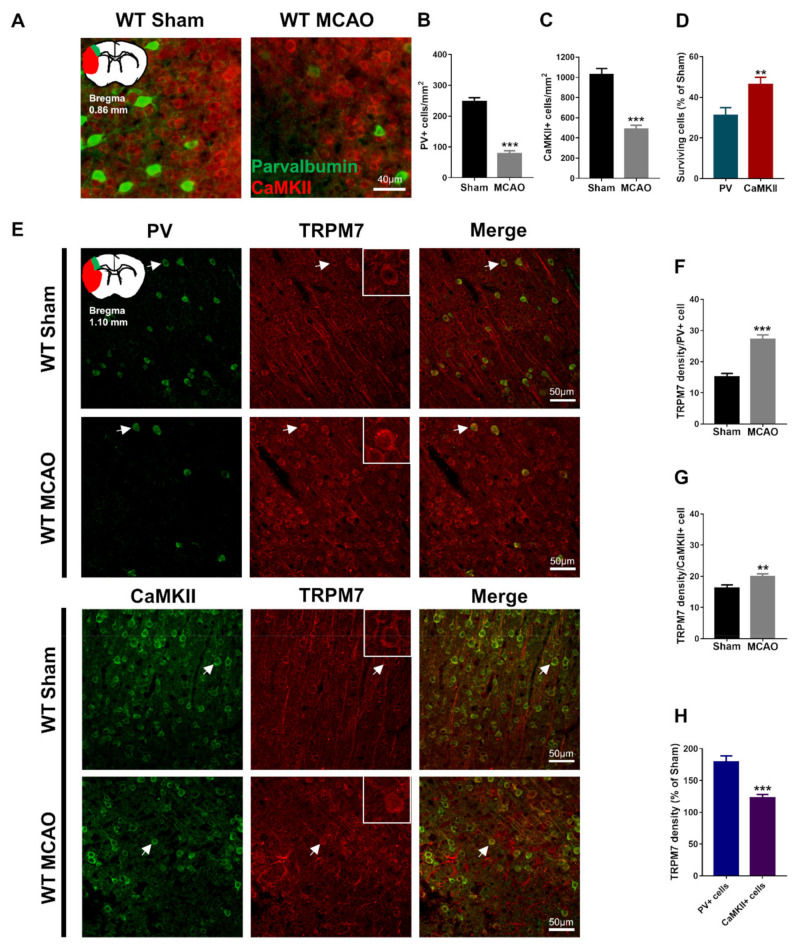
Loss of PV and CaMKII neurons post-brain ischemia and upregulation of TRPM7 expression in different neurons. (**A**) Representative images of PV and CaMKII co-immunostaining in the cortex, and an illustration of the ischemic core (red) and analyzed areas where the images were taken in penumbra (green). (**B**) Quantitative analysis of the number of PV-positive cells. (**C**) Quantitative analysis of the number of CaMKII-positive cells. (**D**) Quantitative analysis of the surviving PV- and CaMKII-positive cells expressed as percentages of respective sham control. (**E**) Representative images of PV and TRPM7 or CaMKII and TRPM7 co-immunostaining in the cortex, and an illustration of the analyzed areas (color-coded as green); examples of co-localization indicated by arrows; (**F**) Quantitative analysis of the TRPM7 density in PV-positive cells. (**G**) Quantitative analysis of the TRPM7 density in CaMKII-positive cells. (**H**) Percentage (%) of the TRPM7 density in PV and CaMKII-positive cells (as a percentage of corresponding sham). Data were obtained from *n* = 54 (**A**–**D**) or *n* = 36 (**E**–**H**) images per group from 3 brain sections per mouse from 6 mice per group. ** *p* < 0.01, *** *p* < 0.001.

Studies provided evidence that GABAergic PV neurons are more sensitive to brain ischemia damage than glutamatergic neurons; however, other studies claimed the opposite [15,16]. We evaluated the number of surviving GABAergic PV neurons and glutamatergic CaMKII neurons in the penumbra of the cortex 7 days after inducing MCAO. Our results showed that PV (Mann–Whitney test, *U* = 153.5, *p* < 0.001; Figure 1A,B) and CaMKII-positive cells (unpaired *t*-test with Welch’s correction, *t*_(90.09)_ = 7.58, *p* < 0.001; Figure 1A,C) were significantly reduced in the MCAO group. Furthermore, we found that the percentage of surviving PV-positive cells was significantly lower than that of the surviving CaMKII-positive cells post-brain ischemia (both were calculated as percentages of corresponding sham control, Mann–Whitney test, *U* = 961, *p* = 0.002; Figure 1D). Our results suggest that GABAergic PV neurons in the cortex are more vulnerable to ischemia-induced neuronal death than glutamatergic neurons.

### 3.2. Brain Ischemia-Induced Upregulation of TRPM7 Is Higher in PV Neurons than in CaMKII Neurons

TRPM7 expression is upregulated following brain ischemia [21]. However, previous studies did not investigate this upregulation in GABAergic vs. glutamatergic neurons. We evaluated the expression of TRPM7 in GABAergic PV neurons and glutamatergic CaMKII neurons in the penumbra of the cortex 7 days after inducing MCAO. Our results showed that TRPM7 expression was significantly higher in PV (unpaired *t*-test, *t*_(70)_ = 6.582, *p* < 0.001; Figure 1E,F) and CaMKII neurons (unpaired *t*-test, *t*_(70)_ = 2.777, *p* = 0.007; Figure 1E,G) of the MCAO group. Thus, brain ischemia resulted in upregulation of TRPM7 in PV and CaMKII neurons. Importantly, we found that the percentage of TRPM7 expression in PV neurons was significantly higher than that in CaMKII neurons (both were calculated as percentages of corresponding sham control, unpaired *t*-test with Welch’s correction, *t*_(54.69)_ = 5.128, *p* < 0.001; Figure 1H). These results suggested that brain ischemia upregulates TRPM7 expression in PV neurons more pronouncedly than in CaMKII neurons.

### 3.3. Knocking out TRPM7 in PV Neurons Has Better Protective Effects against Brain Ischemia than in CaMKII Neurons

**Figure 2:**
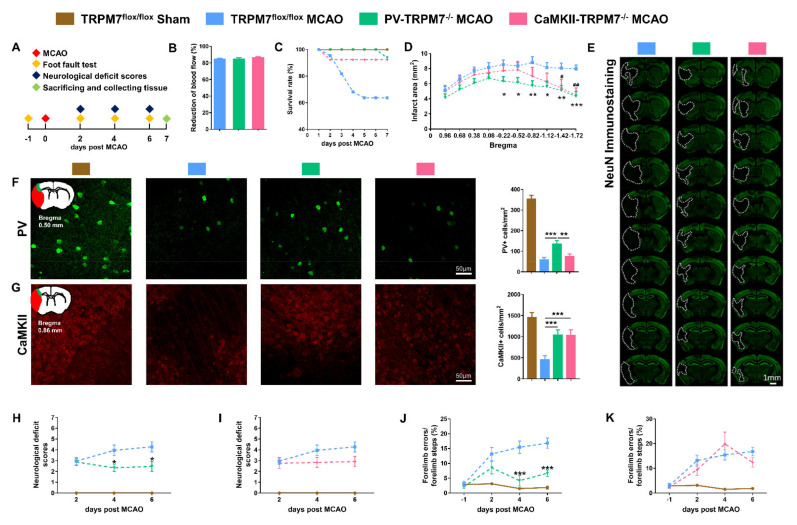
PV-TRPM7^−/−^ protects neurons from ischemic damage and improves the motor ability post-MCAO. (**A**) A schematic diagram of the experimental design. (**B**) Percentage of (%) reduction of blood flow during MCAO procedure, *n* = 22 mice in the TRPM7^flox/flox^ MCAO group, *n* = 15 mice in the PV-TRPM7^−/−^ MCAO group, *n* = 12 mice in the CaMKII-TRPM7^−/−^ MCAO group. (**C**) Survival rate (as percentage, %) of the experimental mice at different post-MCAO time points (days). (**D**) Quantitative analysis of the infarct area, *n* = 14 mice in the TRPM7^flox/flox^ MCAO and PV-TRPM7^−/−^ MCAO groups, *n* = 11 mice in the CaMKII-TRPM7^−/−^ MCAO group; Bonferroni’s post-hoc test. (**E**) Representative NeuN immunostaining images of infarct at 7 days post-MCAO; the white dashed lines indicate the damaged area. (**F**) Representative PV immunostaining images of the cortex, an illustration of the ischemic core (red) and analyzed areas where the images were taken in penumbra (green) and quantitative analysis of the number of PV-positive cells; *n* = 36 images for each group (6 mice per group, 3 brain slices per mouse); Benjamini post-hoc test. (**G**) Representative CaMKII immunostaining images of the cortex, an illustration of the ischemic core (red) and analyzed areas where the images were taken in penumbra (green) and quantitative analysis of the number of CaMKII-positive cells; *n* = 36 images for each group; Benjamini post-hoc test. (**H**,**I**) Neurological deficit scores at different time points (days) post-MCAO; *n* = 7 mice in the TRPM7^flox/flox^ Sham group, *n* = 22 mice in the TRPM7^flox/flox^ MCAO group, *n* = 15 mice in the PV-TRPM7^−/−^ MCAO group (**H**), *n* = 12 mice in the CaMKII-TRPM7^−/−^ MCAO group (**I**–**K**). Foot fault test at different time points (days) post-MCAO; *n* = 7 mice in the TRPM7^flox/flox^ Sham group, *n* = 14 mice in the TRPM7^flox/flox^ MCAO group, *n* = 14 mice for PV-TRPM7^−/−^ MCAO group (**J**), *n* = 11 mice in the CaMKII-TRPM7^−/−^ MCAO group (**K**). Bonferroni’s post-hoc test. TRPM7^flox/flox^ Sham was used as reference but not statistically compared with the other groups. * *p* < 0.05, ** *p* < 0.01, *** *p* < 0.001 (PV-TRPM7^−/−^ MCAO vs. TRPM7^flox/flox^ MCAO), **^#^** *p* < 0.05, **^##^** *p* < 0.01 (CaMKII-TRPM7^−/−^ MCAO vs. TRPM7^flox/flox^ MCAO).

To compare the neuroprotective effects of TRPM7 deletion in PV neurons with that in CaMKII neurons, we generated two lines of conditional knockout mice, PV-TRPM7^−/−^ and CaMKII-TRPM7^−/−^ mice. We confirmed the cell-specific knockout of TRPM7 by crossing PV-TRPM7^−/−^ or CaMKII-TRPM7^−/−^ mice with Ai3 Cre-reporter mice (Supplementary Figure S1A,B and Supplementary Experimental Procedures). Moreover, we confirmed that TRPM7 knockout had no effects on body weight (Supplementary Figure S1C) or motor functions (Supplementary Figure S1D). At 1 day before MCAO, we performed foot fault test for all experimental groups to establish a baseline. At day 2, 4 and 6 post-MCAO, neurological deficit score and foot fault tests were performed. All mice were sacrificed on day 7. All histological, biochemical and cellular analyses were carried out afterwards by using Western blot, ELISA, immunostaining and imaging (Figure 2A). Before and during the occlusion process, we monitored blood flow by laser Doppler flowmetry. We confirmed that our MCAO procedure effectively reduced the blood flow equivalently in all groups by 80% (Figure 2B).

During the 7 days post-ischemia period, we found that TRPM7 knockout in PV (PV-TRPM7^−/−^: 93.8%) and CaMKII (CaMKII-TRPM7^−/−^: 92.3%) neurons resulted in clear enhancement of animals’ survival rate in comparison with that of TRPM7^flox/flox^ MCAO (control group: 63.6%, Figure 2C). To evaluate the post-MCAO brain infarct area, we performed NeuN immunostaining and imaging analysis. We found that the infarct area of PV-TRPM7^−/−^ MCAO mice was significantly lower than that of the TRPM7^flox/flox^ MCAO mice at bregma levels −0.22, −0.52, −0.82 −1.12, −1.42 and −1.72. The value in the CaMKII-TRPM7^−/−^ MCAO mice was significantly reduced in comparison with that of the TRPM7^flox/flox^ MCAO mice at bregma levels −1.42 and −1.72 only (Figure 2D,E; two-way ANOVA repeated measure, effects of brain level: F_(9,324)_ = 23.91, *p* < 0.001; gene knockout: F_(2,36)_ = 3.16, *p* = 0.05 and interaction: F_(18,324)_ = 3.46, *p* < 0.001). Next, we quantified the number of surviving GABAergic PV neurons and glutamatergic CaMKII neurons in the penumbra of the cortex of all the experimental groups. The results revealed that although the PV-TRPM7^−/−^ MCAO mice had a decrease in their PV neuron level in comparison with the sham group, the number of PV-positive cells in these conditional knockout mice was significantly higher than that in the TRPM7^flox/flox^ MCAO and CaMKII-TRPM7^−/−^ MCAO mice (Figure 2F, one-way ANOVA, Kruskal–Wallis test = 14.84, *p* < 0.001). On the other hand, we also found that the PV-TRPM7^−/−^ and CaMKII-TRPM7^−/−^ MCAO mice had significantly higher CaMKII neuron counts than TRPM7^flox/flox^ MCAO mice (Figure 2G; one-way ANOVA, Kruskal–Wallis test = 15.07, *p* < 0.001). Thus, we concluded that both GABAergic PV and glutamatergic neurons are protected by the deletion of TRPM7 in GABAergic PV neurons, but only glutamatergic neurons are protected by the deletion of TRPM7 in glutamatergic neurons after brain ischemia.

At the behavioral level, neurological deficit scores showed that at day 2 both PV-TRPM7^−/−^ MCAO and TRPM7^flox/flox^ MCAO mice had similar deficit scores. At 4 and 6 days post-MCAO, the deficit scores of PV-TRPM7^−/−^ MCAO mice became significantly lower than that of TRPM7^flox/flox^ MCAO mice (Figure 2H; two-way ANOVA repeated measure, effects of time: F_(2,70)_ = 1.22, *p* = 0.303; gene knockout: F_(1,35)_ = 5.21, *p* = 0.028 and interaction: F_(2,70)_ = 5.13, *p* = 0.008). Meanwhile, the deficit scores of CaMKII-TRPM7^−/−^ MCAO mice were not significantly different from these of TRPM7^flox/flox^ MCAO mice (Figure 2I; two-way ANOVA repeated measure, effects of time: F_(2,64)_ = 3.39, *p* = 0.039; gene knockout: F_(1,32)_ = 2.23, *p* = 0.145 and interaction: F_(2,64)_ = 2.13, *p* = 0.127). We also evaluated motor functions by using a foot fault test. Similar to the neurological deficit scores, the percentage of forelimb errors at day 2 did not differ between PV-TRPM7^−/−^ MCAO and TRPM7^flox/flox^ MCAO mice. At 4 and 6 days post-MCAO, the forelimb errors of PV-TRPM7^−/−^ MCAO mice became significantly lower than that of TRPM7^flox/flox^ MCAO mice (Figure 2J; two-way ANOVA repeated measure, effects of time: F_(3,78)_ = 17.4, *p* < 0.001; gene knockout: F_(1,26)_ = 17.63, *p* < 0.001 and interaction: F_(3,78)_ = 5.96, *p* = 0.001). Meanwhile, the forelimb errors of CaMKII-TRPM7^−/−^ MCAO mice were not significantly different from these of TRPM7^flox/flox^ MCAO mice at any time point (Figure 2K; two-way ANOVA repeated measure, effects of time: F_(3,69)_ = 18.78, *p* < 0.001; gene knockout: F_(1,23)_ = 0.303, *p* = 0.588 and interaction: F_(3,69)_ = 1.87, *p* = 0.143). In all experiments, TRPM7^flox/flox^ Sham mice were also evaluated as a reference for normal neurological and motor scores. Therefore, we concluded that ischemia-induced neurological and motor deficits are reduced by the deletion of TRPM7 in GABAergic PV neurons, but not in glutamatergic neurons.

### 3.4. TRPM7 Knockout in PV Neurons Exerts Better Anti-Inflammatory Effects Post-Ischemia

**Figure 3:**
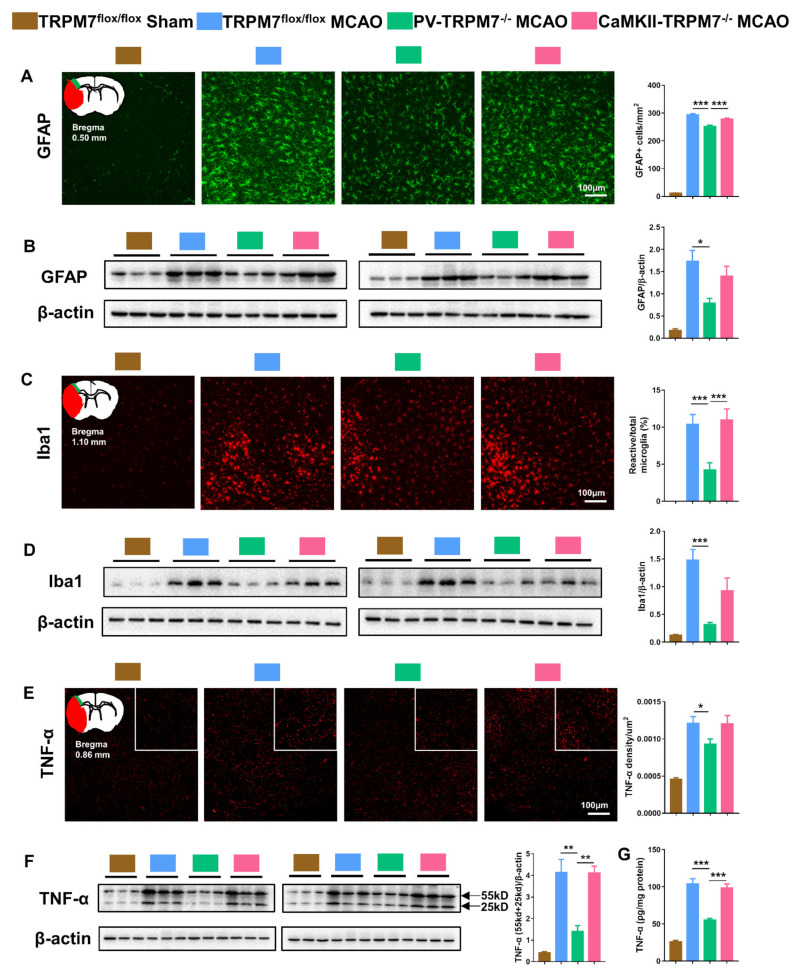
PV-TRPM7^−/−^ has less post-MCAO inflammation. (**A**) Representative GFAP immunostaining images in the cortex, an illustration of the ischemic core (red) and analyzed areas where the images were taken in penumbra (green) and quantitative analysis of the number of GFAP positive cells; Bonferroni’s post-hoc test. (**B**) Western blot images of GFAP and β-actin protein bands and quantitative analysis of GFAP; Bonferroni’s post-hoc test. (**C**) Representative Iba1 immunostaining images in the cortex, an illustration of the ischemic core (red) and analyzed areas where the images were taken in penumbra (green) and quantitative analysis of percentage of reactive microglia; Benjamini post-hoc test. (**D**) Western blot images of Iba1 and β-actin protein bands and quantitative analysis of Iba1; Bonferroni’s post-hoc test. (**E**) Representative TNF-α immunostaining images in the cortex, an illustration of the ischemic core (red) and analyzed areas where the images were taken in penumbra (green) and quantitative analysis of TNF-α density; Benjamini post-hoc test. (**F**) Western blot images of TNF-α (trimeric: 55 kD and dimeric: 25 kD) and β-actin protein bands and quantitative analysis of TNF-α; Bonferroni’s post-hoc test; co-detection of β-actin served as loading and normalizing control. (**G**) Quantitative analysis of TNF-α level by ELISA assay; Bonferroni’s post-hoc test. TRPM7^flox/flox^ Sham was used as reference but not statistically compared with the other groups. Quantitative immunostaining data were obtained from *n* = 54 images per group from 3 brain sections per mouse from 6 mice per group. Western blot and ELISA assay data were obtained from *n* = 6 mice per group. * *p* < 0.05, ** *p* < 0.01, *** *p* < 0.001.

Oxidative stress is well known to be induced by brain ischemia, which is accompanied with inflammation [22,23]. In the present study, we detected related inflammatory factors in the penumbra of the cortex post-MCAO treatment. The results showed that the number of GFAP-positive cells was dramatically higher in all MCAO groups than that in the sham group. However, GFAP-positive cells in PV-TRPM7^−/−^ MCAO mice were significantly decreased in comparison with these in TRPM7^flox/flox^ MCAO and CaMKII-TRPM7^−/−^ MCAO mice (Figure 3A; one-way ANOVA, F_(2,159)_ = 20.87, *p* < 0.001). Western blot analysis supported these data. We found that GFAP protein expression in penumbra was dramatically higher in all MCAO groups than that in the sham group. However, the GFAP level in PV-TRPM7^−/−^ MCAO mice was significantly decreased in comparison with that in TRPM7^flox/flox^ MCAO mice (Figure 3B; one-way ANOVA, F_(2,15)_ = 5.34, *p* = 0.018). Concerning reactive microglia, we observed that the Iba1 expression level was higher in all MCAO groups compared with that in the sham group (Figure 3D). The percentage of reactive microglia (as a percentage of total microglia) in the PV-TRPM7^−/−^ MCAO mice was significantly lower than that in the TRPM7^flox/flox^ MCAO and CaMKII-TRPM7^−/−^ MCAO mice (Figure 3C; one-way ANOVA, Kruskal–Wallis test = 24.13, *p* < 0.001). Protein expression of Iba1 in the PV-TRPM7^−/−^ MCAO mice was also significantly lower than that in the TRPM7^flox/flox^ MCAO mice (Figure 3D; one-way ANOVA, F_(2,15)_ = 11.17, *p* = 0.0011). In all MCAO groups, the expression of TNF-α, which is an important inflammatory factor, was also higher than that in the sham group. Meanwhile, we found that the TNF-α density in the PV-TRPM7^−/−^ MCAO mice was significantly reduced in comparison with that in the TRPM7^flox/flox^ MCAO mice (Figure 3E; one-way ANOVA, Kruskal–Wallis test = 5.77, *p* = 0.05). These data were confirmed by quantitative Western blot (Figure 3F; one-way ANOVA, F_(2,15)_ = 13.56, *p* < 0.001) and ELISA assay (Figure 3G; one-way ANOVA, F_(2,15)_ = 25.06, *p* < 0.001). These results indicated that the deletion of TRPM7 in GABAergic PV neurons, but not in glutamatergic neurons, reduced the post-ischemia inflammatory processes.

### 3.5. Akt/p53/Caspase-3 Signaling Pathways in PV-TRPM7^−/−^ and CaMKII-TRPM7^−/−^ Mice Post-Ischemia

**Figure 4:**
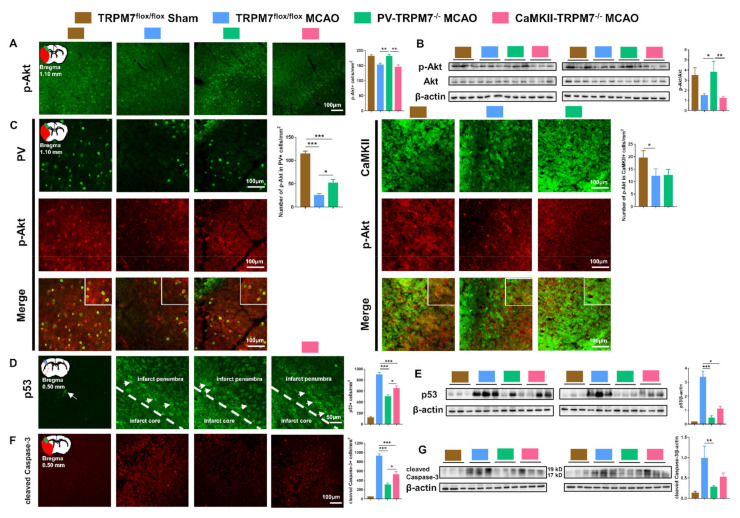
Akt/p53/caspase-3 in PV-TRPM7^−/−^ and CaMKII-TRPM7^−/−^ mice following brain ischemia. (**A**) Representative p-Akt immunostaining images in the cortex, an illustration of the ischemic core (red) and analyzed areas where the images were taken in penumbra (green) and quantitative analysis of the number of p-Akt-positive cells; *n* = 54 images for each group; Benjamini post-hoc test. (**B**) Western blot images of p-Akt, Akt and β-actin protein bands and quantitative analysis of Akt activation expressed as p-Akt/total Akt ratio; Benjamini post-hoc test. (**C**) Representative images of p-Akt and PV or CaMKII co-immunostaining in the cortex, an illustration of the ischemic core (red) and analyzed areas where the images were taken in penumbra (green) and quantitative analysis of the number of p-Akt-/PV-positive (left) or p-Akt-/CaMKII-positive (right) cells; *n* = 36 images for each group; Benjamini post-hoc test. (**D**) Representative p53 immunostaining images (arrows indicate example p53-positive cells), an illustration of the ischemic core (red) and analyzed areas where the images were taken within core/penumbra boundary (blue rectangle) and quantitative analysis of the number of p53-positive cells; *n* = 54 images for each group; Bonferroni’s post-hoc test. (**E**) Western blot images of p53 and β-actin protein bands and quantitative analysis of p53; Benjamini post-hoc test. (**F**) Representative cleaved caspase-3 immunostaining images, an illustration of the ischemic core (red) and analyzed areas where the images were taken in the ischemic core (green) and quantitative analysis of the number of cleaved caspase-3 positive cells; *n* = 54 images for the TRPM7^flox/flox^ Sham group, *n* = 40 images for the TRPM7^flox/flox^ MCAO group, *n* = 49 images for the PV-TRPM7^−/−^ MCAO group, *n* = 43 images for the CaMKII-TRPM7^−/−^ MCAO group; Benjamini post-hoc test. (**G**) Western blot images of cleaved Caspase-3 (cleaved fragments 19 and 17 kD) and β-actin protein bands and quantitative analysis of cleaved Caspase-3; Benjamini post-hoc test. TRPM7^flox/flox^ Sham was used as reference but not statistically compared with the other groups (except for (**C**)); quantitative immunostaining data were obtained from 6 mice per group, 3 brain sections per mouse. Western blot data were obtained from *n* = 6 mice per group; co-detection of β-actin served as normalizing and loading control. * *p* < 0.05, ** *p* < 0.01, *** *p* < 0.001.

Akt signaling has been implicated in ischemia pathologies [24]. Increase in Akt activation (phosphorylation) is associated with enhanced post-ischemia recovery [25]. Therefore, we checked the level of p-Akt in the cortex of all experimental groups and found that the number of p-Akt-positive cells in PV-TRPM7^−/−^ MCAO was significantly higher than that in TRPM7^flox/flox^ MCAO and CaMKII-TRPM7^−/−^ MCAO mice (Figure 4A; one-way ANOVA, Kruskal–Wallis test = 12.36, *p* = 0.002). These results were confirmed by Western blot analysis (Figure 4B; one-way ANOVA, Kruskal–Wallis test = 7.87, *p* = 0.013). Further cell-type-specific analysis revealed that the number of p-Akt-positive cells in PV neurons was significantly higher in PV-TRPM7^−/−^ MCAO mice in comparison with TRPM7^flox/flox^ MCAO, but remained significantly lower than that in sham mice (Figure 4C left; one-way ANOVA, Kruskal–Wallis test = 56.58, *p* < 0.001). Moreover, the number of p-Akt-positive cells in CaMKII neurons was reduced in both PV-TRPM7^−/−^ MCAO and TRPM7^flox/flox^ MCAO (Figure 4C right; one-way ANOVA, Kruskal–Wallis test = 6.32, *p* = 0.042). These results indicated that the deletion of TRPM7 in GABAergic PV neurons, but not glutamatergic neurons, rescued Akt activity in the post-ischemic brain. Furthermore, this rescue is likely to occur in PV neurons and other types of brain cells, but not glutamatergic neurons.

The protein p53 is considered a key regulator of apoptotic processes that are inhibited by Akt [26]. Next, we tested the expression level of p53. We found that the number of p53-positive cells in the PV-TRPM7^−/−^ MCAO mice was significantly lower than that in the TRPM7^flox/flox^ MCAO and CaMKII-TRPM7^−/−^ MCAO mice. Unlike p-Akt data, we found that p53-positive cells were also significantly reduced in CaMKII-TRPM7^−/−^ MCAO in comparison with TRPM7^flox/flox^ MCAO mice (Figure 4D; one-way ANOVA, F_(2,159)_ = 24.12, *p* < 0.001). In line with these data, Western blot analysis revealed that p53 protein expression was significantly lower in PV-TRPM7^−/−^ MCAO and CaMKII-TRPM7^−/−^ MCAO mice in comparison with TRPM7^flox/flox^ MCAO mice (Figure 4E; one-way ANOVA, Kruskal–Wallis test = 13.66, *p* < 0.001). Finally, we detected the expression of a key apoptosis executive factor, namely the cleaved caspase-3, in all experimental groups. Our results showed that the number of cleaved caspase-3 positive cells was significantly reduced in PV-TRPM7^−/−^ MCAO mice in comparison with that in TRPM7^flox/flox^ MCAO and CaMKII-TRPM7^−/−^ MCAO mice. Similar to the p53 results, we found that the cleaved caspase-3 positive cells were also significantly reduced in CaMKII-TRPM7^−/−^ MCAO in comparison with TRPM7^flox/flox^ MCAO mice (Figure 4F; one-way ANOVA, Kruskal–Wallis test = 47.05, *p* < 0.001). A similar pattern was observed by using Western blot analysis (Figure 4G; one-way ANOVA, Kruskal–Wallis test = 9.98, *p* = 0.002). These results indicated that the deletion of TRPM7 in both GABAergic PV neurons and glutamatergic neurons protected brain cells from apoptosis mediating molecules. However, TRPM7 deletion in GABAergic PV neurons activated the anti-oxidative stress/anti-apoptosis Akt signaling.

## 4. Discussion

In the current study, we show that brain ischemia induced higher loss of GABAergic PV neurons than of glutamatergic neurons in the cortex. The ischemia-induced upregulation of TRPM7 expression was found to be higher in GABAergic PV neurons than in glutamatergic neurons, which might explain the sensitivity of GABAergic PV neurons to ischemia-induced neuronal death. In line with the neuroprotective effects of TRPM7 blocking/suppressing, our data show that deleting TRPM7 in PV and/or glutamatergic neurons reduced infarct volume, protected glutamatergic neurons and promoted anti-apoptotic mechanisms. However, we found that knockout of TRPM7 in GABAergic PV neurons was more effective in protecting GABAergic and glutamatergic neurons, promoting neurological and motor recovery, reducing inflammatory processes and inducing anti-apoptosis/anti-oxidative stress signaling pathways.

Both glutamatergic and GABAergic neurons are damaged by cerebral ischemia, but contradictory results have been obtained on their comparative susceptibility to such injuries. Studies suggest that GABAergic interneurons survived the injury for up to 30 days in cortical and hippocampal regions in the rat brain [27]. Pyramidal neurons within CA1 were shown to degenerate at 2 days after ischemia [28]. On the other hand, short-term ischemia permanently impairs the excitability of inhibitory neurons and synaptic transmission mediated by GABA, leading to glutamatergic excitotoxicity [29]. In the current study, we found that both GABAergic PV and glutamatergic neurons are decreased in the cortex following brain ischemia; however, more GABAergic PV neurons were lost in comparison with glutamatergic neurons. Furthermore, the post-ischemia increase in TRPM7 expression was higher in PV neurons than that in glutamatergic neurons. In view of the compelling evidence showing the role of TRPM7 upregulation/hyperactivity in mediating post-ischemia neuronal death [1,2], our results correspond more with previous studies suggesting that ischemia induces more pronounced loss of PV neurons than of glutamatergic neurons [15] and provide a possible explanation for PV neuronal sensitivity, namely the more pronounced upregulation of TRPM7 following ischemia.

Suppression of TRPM7 following brain ischemia reduces neuronal death, infarct volume and motor disability [30]. However, the exact molecular events underlying these neuroprotective effects of TRPM7 suppression remain largely unknown. It is known that excessive Ca^2+^ influx induces oxidative stress and apoptotic cell death [31,32]. Therefore, it is widely believed that, following hypoxia/ischemia, the ion channel part of TRPM7 mediates Ca^2+^ overload, leading to cytotoxicity and neuronal death [1,2]. Studies also suggest that Ca^2+^ overload triggers production of oxygen/nitrogen species, which increases TRPM7-like conductance, leading to further Ca^2+^ toxicity and more neuronal death [6]. On the other hand, TRPM7 suppression reduces Ca^2+^ overload and increases the phosphorylation of endothelial nitric oxide synthase (eNOS), which counteracts the damage caused by oxygen/nitrogen species and hence reduces brain injury [33]. Our data does not contradict this universal (non-cell type specific) mechanism. Preventing Ca^2+^ overload can apply to all cell types, which might explain the observed protective effects of deleting TRPM7 in glutamatergic and GABAergic PV neurons. However, the more pronounced neuroprotective effects of TRPM7 deletion in PV neurons indicate that there are other mechanisms, i.e., cell-type-specific mechanisms.

Akt signaling pathway regulates cell proliferation, growth, differentiation and survival [34,35]. It is implicated in different diseases such as cancer, cardiovascular diseases, diabetes and neurological dysfunctions [24]. Akt is considered one of the key signaling pathways contributing to brain ischemia pathologies and recovery [25]. Several lines of research demonstrate a relationship between Akt signaling pathway and the neuroprotective effects of TRPM7 suppression during brain ischemia. For instance, activation of Akt signaling pathway protects the brain from ischemic injury by downregulating TRPM7 [7,36]. On the other hand, suppression of TRPM7 expression markedly increases the phosphorylation of Akt after brain injury [33]. Moreover, pharmacological suppression of TRPM7 protects neonatal brain from hypoxic-ischemic injury by activating Akt [30]. In line with this mechanism, we found complete restoration (i.e., matching sham level) of Akt activity happened only when TRPM7 was deleted in PV neurons. Furthermore, the Akt activation was found to happen in GABAergic PV, but not glutamatergic neurons. Since activation of Akt in PV neurons did not match the level of sham (Figure 4B), our data suggest that deletion of TRPM7 in PV neurons might also activate Akt in glial cells and other types of GABAergic neurons resulting in the observed complete restoration of the overall activity of Akt (Figure 4A). Downstream from Akt is the apoptosis activating signaling p53/caspase-3 [37]. Attenuation of p53 expression protects against focal ischemic damage in mice [38]. In cell lines exposed to OGD, TRPM7 was found to contribute to activation of p53/caspase-3 apoptotic pathway [39]. Suppression of TRPM7 expression reduces the cleaved caspase-3 in the brain following ischemia in mice [30]. Thus, our data support the link between TRPM7 suppression and activation of Akt under brain ischemia pathologies, but clearly indicate that such activation is dependent on the cell type in which TRPM7 was deleted (i.e., cell-type-specific mechanism). Furthermore, we confirmed the involvement of p53/caspase-3 in the neuroprotective effects of TRPM7 deletion. However, we found that suppression of such pro-apoptosis signaling molecules represents a universal neuroprotective response to TRPM7 deletion in both types of cells. Based on that, one might speculate that the TRPM7-dependent effects on p53/caspase-3 signaling may not necessarily require activation of Akt signaling at least in glutamatergic neurons and that activation of Akt signaling might underlie the stronger neuroprotective effects of deleting TRPM7 in PV neurons.

In conclusion, we found that, in comparison with glutamatergic neurons, knockout of TRPM7 in GABAergic PV neurons has better neuroprotective and/or recovery effects following ischemia as well as stronger activation of anti-oxidative stress signaling within the post-ischemic brain. The results underline the importance of studying TRPM7 roles in brain pathology in a cell-type-specific manner. This should advance our understanding of TRPM7 contributions to brain pathologies as well as our knowledge regarding the molecular mechanisms linked to the channel functions and/or dysfunctions.

## Figures and Tables

**Table 1:** Microscopic, optical and imaging information. NA, numerical aperture of the lens.

Lens	Digital Zoom	Pixels Resolution	Area of the Optical Field	Number of Optical Field	Figure No.
10× (NA 0.40)	1	/	Whole brain section	/	Figure 2E
20× (NA 0.75)	1	1024 × 1024	(635 × 635) μm^2^	3	Figures 3A, 3C, 3E, 4A and 4D
20× (NA 0.75)	1	1240 × 1240	(400 × 400) μm^2^	2	Figure 4B
40× (NA 0.95)	1	1024 × 1024	(317 × 317) μm^2^	2	Figures 1E, 2F and 2G
40× (NA 0.95)	1	1024 × 1024	(317 × 317) μm^2^	3	Figure 4C
40× (NA 0.90)	1	1240 × 1240	(200 × 200) μm^2^	3	Figure 1A

## Data Availability

Data is contained within the article or Supplementary Materials.

## Supplementary Materials

The following are available online at https://www.mdpi.com/article/10.3390/cells11071178/s1, Supplementary Figure S1: Confirmation of PV-TRPM7^−/−^ or CaMKII-TRPM7^−/−^ mice; body weight of experimental animals before MCAO; open-field test before MCAO, Supplementary Experimental Procedures: Immunofluorescence and Open-Field Test; Table S1: Results of normality test and variance homogeneity test of each Figure.
