# Supplementary material for: Neuroprotective Effects of TRPM7 Deletion in Parvalbumin GABAergic vs. Glutamatergic Neurons following Ischemia
Source: Cells. 2022 Mar 31;11(7):1178. doi: 10.3390/cells11071178 (PMC8997982; doi:10.3390/cells11071178)
Supplement: Supplementary file 1 [file cells-11-01178-s001.zip › cells-1654451-supplementary.pdf]

cells

Supplementary Materials

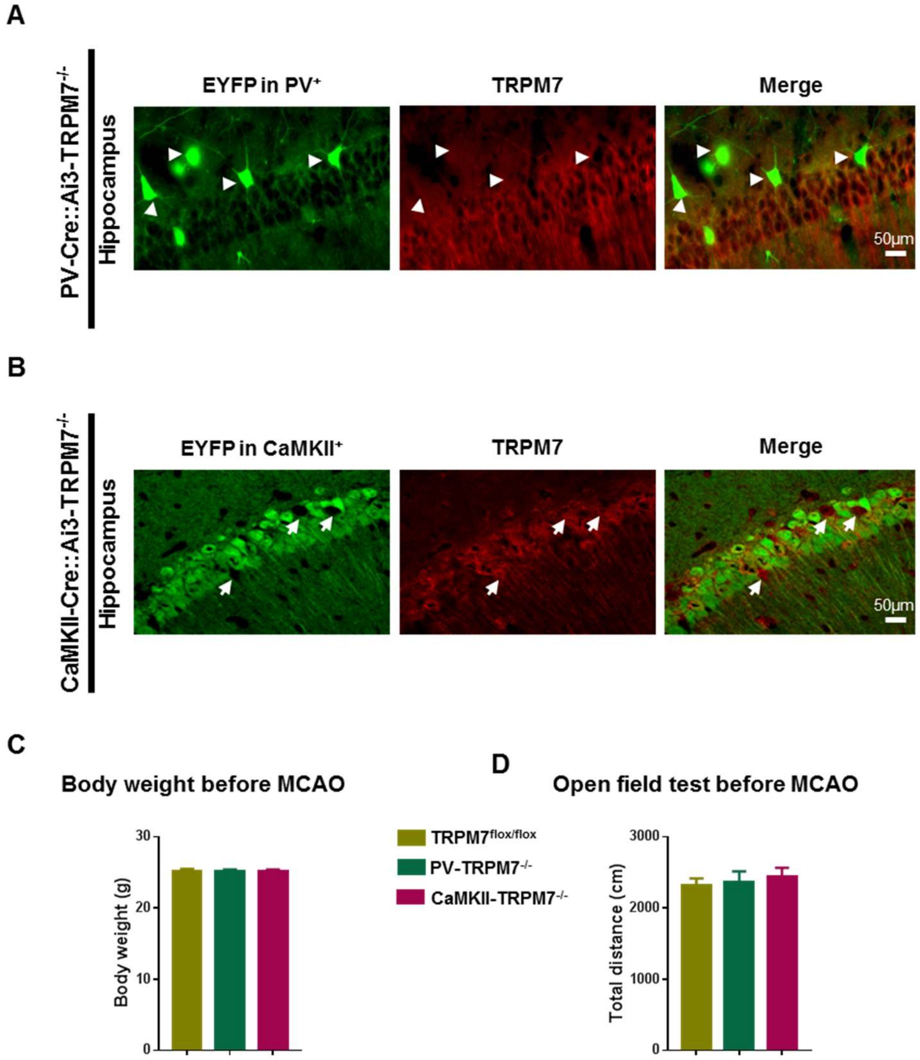

Figure S1. Confirmation of PV-TRPM7$^{-/-}$ or CaMKII-TRPM7$^{-/-}$ mice; body weight of experimental animals before MCAO; open-field test before MCAO. (**A**) Representative fluorescent images of PV-Cre::Ai3-TRPM7$^{-/-}$ brain sections stained with TRPM7 in the hippocampus. Arrows indicate the PV neurons did not have TRPM7 signal; (**B**) Representative fluorescent images of CaMKII-Cre::Ai3-TRPM7$^{-/-}$ brain sections stained with TRPM7 in the hippocampus. Arrows indicate the TRPM7 positive cells not co-localized with CaMKII neurons; (**C**) Quantitative analysis of the body weight before MCAO. n = 14 mice for the TRPM7$^{flox/flox}$ group, n = 15 mice for the PV-TRPM7$^{-/-}$ group, and n = 12 mice for the CaMKII-TRPM7$^{-/-}$ group; (**D**) Open-field test before MCAO. n = 5 mice for the TRPM7$^{flox/flox}$ group, n = 6 mice for PV-TRPM7$^{-/-}$ group, n = 7 mice for CaMKII-TRPM7$^{-/-}$ group. Data suggest the different genetic backgrounds did not affect locomotor activity in mice. All aforementioned data are expressed as mean ± SEM.

Citation: Zhang, P.; Li, W.; Liu, Y.; Gao, Y.; Abumaria, N. Neuroprotective Effects of TRPM7 Deletion in Parvalbumin GABAergic vs. Glutamatergic Neurons following Ischemia. *Cells* **2022**, *11*, 1178. https://doi.org/10.3390/cells11071178

Academic Editors: Lin-Hua Jiang, Xiaoqiang Yao and Bilal ÇİĞ

Received: 11 March 2022
Accepted: 29 March 2022
Published: 31 March 2022

Publisher's Note: MDPI stays neutral with regard to jurisdictional claims in published maps and institutional affiliations.

Copyright: © 2022 by the authors. Licensee MDPI, Basel, Switzerland. This article is an open access article distributed under the terms and conditions of the Creative Commons Attribution (CC BY) license (https://creativecommons.org/licenses/by/4.0/).

Supplementary Experimental Procedures

Immunofluorescence

PV-TRPM7$^{-/-}$ and CaMKII-TRPM7$^{-/-}$ mice were crossed with Ai3 reporter mice to generate PV-Cre::Ai3-TRPM7$^{-/-}$ and CaMKII-Cre::Ai3-TRPM7$^{-/-}$ mice expressing EYFP in GABAergic PV and glutamatergic CaMKII neurons (respectively). The PV-Cre::Ai3-TRPM7$^{-/-}$ and CaMKII-Cre::Ai3-TRPM7$^{-/-}$ mice were sacrificed under anesthesia and perfused with 4% paraformaldehyde. After gradient dehydration with 10%, 20%, and 30% sucrose, brain tissues were embedded in an optimum cutting temperature compound (OCT) tissue-freezing medium. Then, 25-μm frozen sections were prepared. All sections were blocked with blocking solution (5% goat serum and 0.3% Triton X-100) for 90 min at room temperature. Next, the sections were incubated overnight at 4 °C with the primary antibody of anti-TRPM7 (1:100, Alomone). After washing with 0.01 M PBS, sections were incubated with CF-647 (1:500, Biotium) conjugated secondary antibody for 2 h at room temperature and the stained sections were photographed using confocal microscopy systems (FV1000, Olympus).

Open-Field Test

An open-field test was performed without ischemia exposure. Mice were placed in a 50 × 50 × 50 cm instrument for 5 min. Sessions were recorded and videos were analyzed by using Lime Light software (Coulbourn, USA).

Table S1. Results of normality test and variance homogeneity test of each Figure. $p < 0.05$ means that the data do not meet normality or homogeneity of variance. NA, not applicable.

Figure No.	Results of Shapiro-Wilk Normality Test Group and p-Value			Results of Variance Homogeneity Test (F Test)	
Figure 1B	Sham<br>$p = 0.0638$	MCAO<br>$p < 0.001$	/<br>/	NA	NA
Figure 1C	Sham<br>$p = 0.7952$	MCAO<br>$p = 0.1185$	/<br>/	F(53,53) = 2.449	$p = 0.0014$
Figure 1D	PV<br>$p < 0.001$	CaMKII<br>$p = 0.1185$	/<br>/	NA	NA
Figure 1F	Sham<br>$p = 0.7842$	MCAO<br>$p = 0.5075$	/<br>/	F(35,35) = 1.802	$p = 0.0858$
Figure 1G	Sham<br>$p = 0.0860$	MCAO<br>$p = 0.5324$	/<br>/	F(35,35) = 1.313	$p = 0.4248$
Figure 1H	PV+ cells<br>$p = 0.5075$	CaMKII+ cells<br>$p = 0.5324$	/<br>/	F(35,35) = 3.248	$p < 0.001$
Figure 2F	TRPM7$^{flox/flox}$ MCAO<br>$p < 0.001$	PV-TRPM7$^{-/-}$ MCAO<br>$p = 0.0014$	CaMKII-TRPM7$^{-/-}$ MCAO<br>$p < 0.001$	NA	NA
Figure 2G	TRPM7$^{flox/flox}$ MCAO<br>$p < 0.001$	PV-TRPM7$^{-/-}$ MCAO<br>$p = 0.0928$	CaMKII-TRPM7$^{-/-}$ MCAO<br>$p = 0.0192$	NA	NA
Figure 3A	TRPM7$^{flox/flox}$ MCAO<br>$p = 0.4458$	PV-TRPM7$^{-/-}$ MCAO<br>$p = 0.4353$	CaMKII-TRPM7$^{-/-}$ MCAO<br>$p = 0.3673$	F(2,159) = 1.994	$p = 0.1395$
Figure 3B	TRPM7$^{flox/flox}$ MCAO<br>$p = 0.2828$	PV-TRPM7$^{-/-}$ MCAO<br>$p = 0.1559$	CaMKII-TRPM7$^{-/-}$ MCAO<br>$p = 0.7749$	F(2,15) = 1.081	$p = 0.3644$
Figure 3C	TRPM7$^{flox/flox}$ MCAO<br>$p < 0.001$	PV-TRPM7$^{-/-}$ MCAO<br>$p < 0.001$	CaMKII-TRPM7$^{-/-}$ MCAO<br>$p < 0.001$	NA	NA
Figure 3D	TRPM7$^{flox/flox}$ MCAO<br>$p = 0.8775$	PV-TRPM7$^{-/-}$ MCAO<br>$p = 0.9538$	CaMKII-TRPM7$^{-/-}$ MCAO<br>$p = 0.5751$	F(2,15) = 2.679	$p = 0.1012$
Figure 3E	TRPM7$^{flox/flox}$ MCAO<br>$p < 0.001$	PV-TRPM7$^{-/-}$ MCAO<br>$p = 0.0069$	CaMKII-TRPM7$^{-/-}$ MCAO<br>$p < 0.001$	NA	NA
Figure 3F	TRPM7$^{flox/flox}$ MCAO<br>$p = 0.1073$	PV-TRPM7$^{-/-}$ MCAO<br>$p = 0.3445$	CaMKII-TRPM7$^{-/-}$ MCAO<br>$p = 0.3764$	F(2,15) = 0.8032	$p = 0.4662$
Figure 3G	TRPM7$^{flox/flox}$ MCAO<br>$p = 0.6246$	PV-TRPM7$^{-/-}$ MCAO<br>$p = 0.1134$	CaMKII-TRPM7$^{-/-}$ MCAO<br>$p = 0.2300$	F(2,15) = 3.221	$p = 0.0686$
Figure 4A	TRPM7$^{flox/flox}$ MCAO<br>$p < 0.001$	PV-TRPM7$^{-/-}$ MCAO<br>$p = 0.1699$	CaMKII-TRPM7$^{-/-}$ MCAO<br>$p = 0.0245$	NA	NA
Figure 4B	TRPM7$^{flox/flox}$ MCAO<br>$p = 0.8299$	PV-TRPM7$^{-/-}$ MCAO<br>$p = 0.0932$	CaMKII-TRPM7$^{-/-}$ MCAO<br>$p = 0.0260$	NA	NA
Figure 4C left	TRPM7$^{flox/flox}$ Sham<br>$p = 0.7433$	TRPM7$^{flox/flox}$ MCAO<br>$p < 0.001$	PV-TRPM7$^{-/-}$ MCAO<br>$p < 0.001$	NA	NA

Figure 4C right	TRPM7$^{flox/flox}$ Sham<br>*p* = 0.0018	TRPM7$^{flox/flox}$ MCAO<br>*p* < 0.001	PV-TRPM7$^{-/-}$ MCAO<br>*p* < 0.001	NA	NA
Figure 4D	TRPM7$^{flox/flox}$ MCAO<br>*p* = 0.0760	PV-TRPM7$^{-/-}$ MCAO<br>*p* = 0.5786	CaMKII-TRPM7$^{-/-}$ MCAO<br>*p* = 0.4288	F(2,159) = 3.172	*p* = 0.0446
Figure 4E	TRPM7$^{flox/flox}$ MCAO<br>*p* = 0.3161	PV-TRPM7$^{-/-}$ MCAO<br>*p* = 0.0035	CaMKII-TRPM7$^{-/-}$ MCAO<br>*p* = 0.4105	NA	NA
Figure 4F	TRPM7$^{flox/flox}$ MCAO<br>*p* = 0.4185	PV-TRPM7$^{-/-}$ MCAO<br>*p* < 0.001	CaMKII-TRPM7$^{-/-}$ MCAO<br>*p* < 0.001	NA	NA
Figure 4G	TRPM7$^{flox/flox}$ MCAO<br>*p* = 0.0399	PV-TRPM7$^{-/-}$ MCAO<br>*p* = 0.1302	CaMKII-TRPM7$^{-/-}$ MCAO<br>*p* = 0.2475	NA	NA
